# A cross-sectional study on the current status and influencing factors of core competencies among public health students

**DOI:** 10.3389/fpubh.2026.1826592

**Published:** 2026-07-01

**Authors:** Bin Liu, Daikun Zheng, Li Shen, Li Liu, Huiye Tan, Zhen Li

**Affiliations:** 1 Department of Public Health and Management, Chongqing Three Gorges Medical College, Chongqing, China; 2 Chongqing Three Gorges Medical College, Chongqing, China; 3 The Affiliated Yongchuan Hospital of Chongqing Medical University, Chongqing, China; 4 Institute of Higher Education Evaluation, Chongqing Educational Evaluation Institute, Chongqing, China

**Keywords:** core competencies, education, internal differences, learning environment perception, public health students

## Abstract

**Background:**

The core competencies of public health students represent the essential character and key capabilities they must possess to adapt to lifelong development and meet societal needs. These competencies directly influence the effectiveness of public health work, such as emergency response and health promotion. Therefore, this study aims to assess the core competency scores of public health students and identify their influencing factors.

**Methods:**

An online cross-sectional study was conducted in January 2026. Data on demographic characteristics, core competencies, and learning environment perception were collected through questionnaires. Independent samples *t*-tests and one-way ANOVA were used to compare internal differences among students with different characteristics, and multiple linear regression was employed to explore the influence of different dimensions of learning environment perception on core competencies.

**Results:**

A total of 271 valid questionnaires were collected. The core competency score of public health students was 4.06 ± 0.49. Awareness of core competencies was positively correlated with core competency scores. No significant differences in core competency scores were observed by gender, ethnicity, household registration, major, grade level, or student leadership experience. Multiple linear regression showed that peer relationships (β = 0.52, *P* < 0.01), project practice (β = 0.33, *P* < 0.01), and learning atmosphere (β = 0.16, *P* < 0.01) had significant positive predictive effects on core competencies, collectively explaining 76.5% of the variance.

**Conclusion:**

The overall core competencies of public health students are at a relatively good level, and their awareness of core competencies is closely related to their actual performance. Key factors influencing core competencies include peer relationships, project practice, and learning atmosphere within the learning environment. It is recommended to enhance publicity and education on core competencies, optimize the design of the learning environment, and emphasize the integration of humanistic education with professional practice, in order to systematically improve the comprehensive competencies of public health talents.

## Background

The European Union defines core competencies as those required for individuals to become active citizens, integrate into society, achieve successful employment, and fulfill self-realization (1). The definition of core competencies in the United States mainly refers to the qualities that students and social practitioners should possess to support further study or social employment (2). In China, the Ministry of Education, in collaboration with universities, has developed the *Core Competencies for Chinese Student Development* framework (3), *The Guiding Opinions on Accelerating the Innovative Development of Medical Education* was released by the General Office of the State Council (4, 5). The Core Competencies for Public Health Professionals represent foundational or crosscutting knowledge and skills for the broad practice of public health that professionals may want to possess as they work to protect and improve the nation's health (6). Core competencies, as the key capabilities and essential character traits required for individuals to adapt to lifelong development and societal needs, have become a central focus in global education reform and talent cultivation (7, 8).

Core competencies are essential for public health students. Engaged in public health prevention, health promotion and emergency response, they need integrated abilities to adapt to professional demands and undertake public health services (9, 10). Against the background of the New Medical Science education reform, existing researches on core competencies mainly focus on general college students or clinical medical students. At present, few studies have explored the intra-group differences in core competencies among public health students as well as the impacts of learning environments on them. Meanwhile, we draw on the medical core competency system, since clinical medicine is an important component of the public health system. Though public health students will work in diverse workplaces instead of only clinical medical institutions. Nevertheless, basic medical knowledge, professional ethics and competency standards are the fundamental foundation for their future work. Referencing the well-established framework of medical core competencies can effectively standardize the competency evaluation and training of public health students. So this study took public health students as the research subjects and analyzed their core competency levels and differences across various demographic characteristics through a questionnaire survey. According to the institutional impact theory (11–13), the institutional environment exerts an influence on students' development. To further explore the impact of school-related factors on the core competencies of public health students, this study also investigated these students' perceptions of the learning environment and examined the relationship between perceived learning environment and their core competencies. We expect that these findings will provide empirical evidence for improving the core competencies of public health students, and offer implications for cultivating a high-caliber workforce of public health professionals.

## Methods

### Sampling and participants

From January to February 2026, a web-based cross-sectional study was conducted. Using a random cluster sampling method, public health students from a medical university in China were selected as study participants (7). Data were collected through self-reported scales, and participants were required to complete the questionnaire independently. Questionnaires were considered valid only if the completion rate for the main sections reached 90% or higher. All null or blank responses were excluded from the final analysis (14). A total of 306 individuals participated in the questionnaire survey, among which 271 were valid.

### Questionnaire

The questionnaire was independently designed by our research team. We referenced the research outcomes of scholars including Fengqiong Liu (15), Sudip Bhandari (16), Judith G. Calhoun (17). We also examined existing core competency frameworks from Canada, the UK, Europe, New Zealand, and the Americas (18–21). These frameworks were translated, revised, and culturally adapted for application among Chinese students. Finally, building upon the *Core Literacy of Chinese Student Development* framework, we developed the core competencies questionnaire. The questionnaire covers six categories, measured through 43 self-report items. The skills assessed by this scale include: (1) Humanistic Foundation, (2) Scientific Spirit, (3) Learning to Learn, (4) Healthy Living, (5) Responsibility and Commitment, and (6) Practical Innovation. Responses are recorded on a 5-point Likert scale, ranging from 5 (“Very Consistent”) to 1 (“Very Inconsistent”). The Cronbach's α coefficient for the scale was 0.973.

The *Learning Environment Perception Questionnaire* was adapted from the scale developed by Li Chen (22). It covers the main aspects of the learning environment that students perceive as influential to their learning and development, and consists of six dimensions with a total of 26 items: (1) Peer Relationships, (2) Course Learning, (3) Learning Atmosphere, (4) Teacher-Student Relationships, (5) Institutional Environment, and (6) Project Practice. It employs a 5-point Likert scale, ranging from 5 (“Very Satisfied”) to 1 (“Very Dissatisfied”). The Cronbach's α coefficient for the scale was 0.971.

### Data analysis

All data were analyzed using SPSS Statistics 29.0 (IBM Corp. IBM SPSS Statistics for MAC, Version 29.0. Armonk, NY: IBM Corp.). The software was utilized to conduct descriptive and correlational analyses. Continuous variables are presented as means and standard deviations (SD), while categorical variables are presented as absolute and relative frequencies Major, grade level, gender, ethnicity, household registration status, student leadership experience, and awareness of core competencies were treated as independent variables (IVs). Differences among groups were analyzed using t-tests and analysis of variance (ANOVA) (23).

A multiple linear regression was conducted to identify variables independently associated with core competencies when accounting for perceptions of the learning environment (*P* < 0.05).

## Results

### Characteristics of the sample

Demographic data included major, grade, gender, ethnicity, household registration, student leadership experience, and awareness of core competencies. Descriptive statistical methods were used for analysis, the characteristics are as follows: students majoring in Preventive Medicine accounted for the highest proportion (41.3%, *n* = 113), followed by Public Health Management (20.7%, *n* = 56), Health Information Management (28.4%, *n* = 77), Food Nutrition and Health (3.3%, *n* = 9), and Health Big Data Management and Service (6.3%, *n* = 17). Sophomores constituted the main surveyed group (55.7%, *n* = 151), with juniors accounting for 27.7% (*n* = 75) and freshmen for 16.6% (*n* = 45). Female students (73.8%) significantly outnumbered male students (26.2%). Han ethnicity students formed the majority (83.4%, *n* = 226), while ethnic minority students accounted for 16.6% (*n* = 45). Students with rural household registration (71.6%) represented a higher proportion than those with urban registration (28.4%). Students with student leadership experience made up 52.4% (*n* = 142).

### Core competency level

The mean score for public health students' core competencies was 4.06 (SD = 0.49). The scores ranged from 2.56 to 5. The highest score was reported for *Responsibility and Commitment* (mean = 4.16, SD = 0.53), while *Humanistic Foundation* had the lowest subscale score (mean = 3.95, SD = 0.59). Scores in the dimensions of Learning to Learn, Healthy Living, and Practical Innovation were slightly below the overall mean score (See Table 1).

**Table 1:** Scores of core competency categories.

Subscale	Items	Minimum	Maximum	Mean	SD	Mean ±SD
Humanistic foundation	I often read books in fields such as literature, history, and philosophy. As a result, I have accumulated a certain amount of humanistic knowledge	1	5	3.44	0.968	3.95 ± 0.589
	I understand the basic meaning of the humanistic spirit. I also agree with the ideas it represents	1	5	3.73	0.787	
	I understand the principle of putting people first. I respect and uphold human dignity and value	1	5	4.30	0.686	
	I can show concern for human existence, development, and wellbeing	1	5	4.14	0.659	
	I have a basic ability to appreciate art. I also hold a healthy aesthetic value	1	5	4.12	0.700	
Scientific spirit	I advocate for science and truth. I can understand and apply basic scientific principles and methods	2	5	4.08	0.656	4.13 ± 0.512
	I respect facts and maintain a diligent attitude toward acquiring knowledge	2	5	4.20	0.560	
	I can think and make judgments independently. I do not blindly follow what others say or do	2	5	4.13	0.602	
	I can analyze issues dialectically and consider them from multiple perspectives. This helps me make appropriate choices	2	5	4.10	0.594	
	I am a curious and imaginative person	2	5	4.15	0.599	
	When facing problems, I actively search for effective solutions	2	5	4.11	0.557	
Learning to learn	I maintain a positive attitude toward learning and have a strong interest in it	1	5	3.96	0.739	4.03 ± 0.570
	I have good learning habits and have developed a set of study methods that suit me	1	5	3.86	0.792	
	I can regularly reflect on my learning progress and summarize what I have achieved	2	5	3.89	0.760	
	If my learning outcomes are not satisfactory, I can adjust my mindset and study methods in time	1	5	3.92	0.721	
	I can fully accept and adapt to the “Internet+” era. I know how to correctly access and use information, and I do not casually disclose personal information online	2	5	4.19	0.609	
	I also adhere to basic online ethics and morality when using the internet. I do not believe or spread rumors, nor do I post inappropriate comments	2	5	4.36	0.592	
Healthy living	I exercise regularly, maintain a balanced diet, and follow a consistent daily routine	1	5	3.87	0.815	4.06 ± 0.571
	I am safety-conscious and possess the awareness and ability to protect myself	2	5	4.26	0.571	
	I am confident and optimistic. Even when facing setbacks or challenges, I do not give up easily	1	5	4.07	0.634	
	I can effectively regulate and manage my emotions	1	5	4.08	0.645	
	I understand my own strengths and weaknesses and have a clear self-awareness	2	5	4.08	0.636	
	I can manage my time wisely and balance the demands of study, leisure, work, and other areas	2	5	3.97	0.725	
Responsibility and commitment	In daily life, I am civilized and polite. I am tolerant toward others and act with integrity	2	5	4.24	0.583	4.16 ± 0.525
	I abide by laws and regulations and actively participate in public welfare activities and volunteer services	2	5	4.15	0.595	
	I appreciate the outstanding cultural achievements and traditional heritage of the Chinese nation. I am willing to inherit and promote these accomplishments	2	5	4.25	0.605	
	I am open to embracing outstanding cultures from other countries. I respect the diversity and differences among cultures	2	5	4.17	0.611	
	I care about global development trends and pay attention to the major challenges facing human	2	5	4.03	0.682	
Practical innovation	I understand the value of respecting the fruits of others' labor. I participate in activities such as internships and part-time jobs during my spare time	2	5	4.10	0.682	4.03 ± 0.590
	I have strong hands-on skills and have mastered some basic labor skills	1	5	4.10	0.619	
	I can often identify problems in my studies and daily life. I am willing to solve these problems	2	5	4.04	0.631	
	I can analyze specific problems on a case-by-case basis and develop appropriate solutions	1	5	4.04	0.674	
Total		2.56	5.00	4.06	0.494

Based on the performance of individual survey items: In the *Humanistic Foundation* category, items such as “Respecting and upholding human dignity and value” (4.30 points) and “Showing concern for human survival, development, and wellbeing” (4.14 points) scored relatively high, reflecting students' strong awareness of humanistic care. However, “Reading literature, history, and philosophy books” (3.44 points) and “Understanding the core meaning of humanistic spirit” (3.73 points) scored relatively low, indicating comparatively weak accumulation of humanistic knowledge and theoretical understanding.

In the *Scientific Spirit* category, items such as “Respecting facts and pursuing knowledge rigorously” (4.20 points) and “Thinking independently without blind conformity” (4.13 points) performed notably well, aligning closely with the scientific thinking requirements of public health education. The variation in scores across items within this category was small (standard deviation 0.51), showing balanced overall performance.

In the *Learning to Learn* category, “Adhere to online ethics and morality” (4.36 points) and “Actively seeking solutions to problems” (4.11 points) led in scores, reflecting students' strong sense of social responsibility and problem awareness. However, “Adaptability of learning habits and methods” (3.86 points) scored relatively low, indicating insufficient development of self-directed learning ability.

In the *Healthy Living* category, “Awareness of safety and self-protection” (4.26 points) and “Normative use of online information” (4.19 points) showed good results, suggesting students have sound physical and mental self-management and digital literacy. However, “Regular exercise and routine” (3.87 points) scored relatively lower, indicating insufficient development of healthy lifestyles.

In the *Responsibility and Accountability* category, “Civility, Politeness, and integrity” (4.24 points) and “Inheriting excellent traditional culture” (4.25 points) ranked among the highest, reflecting students' strong social responsibility and cultural identity, making this a strength among the core competencies.

In the *Practical Innovation* category, “Hands-on operational ability” (4.10 points) and “Identifying and solving problems” (4.04 points) showed acceptable performance. However, “Transforming creative ideas into practical products” (3.89 points) scored relatively low, indicating a gap in converting practical ideas into concrete results.

### Comparison of internal differences in core competencies

Taking the total core competency score as the dependent variable, independent samples *t*-tests were conducted with gender, ethnicity, household registration, and student leadership experience as independent variables. One-way analysis of variance (ANOVA) was performed with major, grade level, and awareness of core competencies as independent variables. The results (Table 2) revealed a statistically significant difference (*P* < 0.05) was found in the total core competency scores based on students' awareness of core competencies. The score for the group who reported “Heard of it and understand” was significantly higher than those for the groups who reported “Never heard of it” and “Heard of it, but not familiar”.

**Table 2:** Comparison of total core competency scores among public health students with different characteristics.

Sociodemographic variables		*N* = 271 (%)	Mean	SD	T/F	*p*-value
Gender	Male	71 (26.2%)	4.05	0.54	0.471	0.493
	Female	200 (73.8%)	4.06	0.48		
Grade	Freshman	45 (16.6%)	4.18	0.43	1.673	0.190
	Sophomore	151 (55.7%)	4.03	0.53		
	Junior	75 (27.7%)	4.05	0.45		
Major	Preventive medicine	112 (41.3%)	4.00	0.48	0.964	0.428
	Public health management	56 (20.7%)	4.07	0.48		
	Health information management	77 (28.4%)	4.12	0.52		
	Food nutrition and health	9 (3.3%)	4.21	0.55		
	Health big data management and service	17 (6.3%)	4.09	0.46		
Ethnicity	Han ethnicity	226 (83.4%)	4.05	0.49	0.103	0.749
	Ethnic minority	45 (16.6%)	4.13	0.49		
Household registration	Urban	77 (28.4%)	4.12	0.52	1.098	0.296
	Rural	194 (71.6%)	4.04	0.48		
Student leadership experience	Yes	142 (52.4%)	4.11	0.48	1.552	0.646
	No	129 (47.6%)	4.00	0.51		
Awareness of core competencies	Never heard of it	25 (9.2%)	3.84	0.46	27.132	< 0.001
	Heard of it, but not familiar	169 (62.4%)	3.95	0.42		
	Heard of it and understand it	77 (28.4%)	4.38	0.51		

### Analysis of the influence of learning environment perception on core competencies

A correlation between the dependent and independent variables is required for regression analysis to be valid. Therefore, prior to conducting regression analysis, a correlation analysis was performed between the dependent and independent variables. Pearson correlation analysis showed that core competencies were significantly positively correlated with all six dimensions of learning environment perception (Table 3) (24).

**Table 3:** Correlation between core competencies and learning environment perception.

IV	Core competencies	Peer relationships	Course learning	Learning atmosphere	Teacher-student relationships	Institutional environment	Project practice
Core competencies	1						
Peer relationships	0.810^**^	1					
Course learning	0.690^**^	0.716^**^	1				
Learning atmosphere	0.741^**^	0.722^**^	0.867^**^	1			
Teacher-student relationships	0.722^**^	0.700^**^	0.752^**^	0.866^**^	1		
Institutional environment	0.728^**^	0.687^**^	0.710^**^	0.771^**^	0.847^**^	1	
Project practice	0.717^**^	0.565^**^	0.578^**^	0.643^**^	0.670^**^	0.789^**^	1

^**^Correlation is significant at the 0.01 level (2-tailed).

Taking the core competency score as the dependent variable and the six dimensions of learning environment perception as independent variables, a multiple linear regression analysis was conducted (25, 26). The results show that the model's D-W value is 2.085, close to 2, indicating no autocorrelation in the residuals and a good model fit. All VIF values were below 10, suggesting no severe multicollinearity among the variables, making the data suitable for regression analysis.

The multiple linear regression results indicate that all six dimensions of learning environment perception—course learning (X_1_), peer relationships (X_2_), learning atmosphere (X_3_), teacher-student relationships (X_4_), institutional environment (X_5_), and project practice (X_6_)—entered the regression equation.

The regression model can be expressed as: *Y* = 0.48–0.014X_1_ + 0.472X_2_ + 0.138X_3_ + 0.037X_4_—0.03X_5_ + 0.278X_6_

This model explains 76.7% of the variance in core competencies. Among the dimensions, peer relationships (β = 0.510, *P* < 0.001), project practice (β = 0.333, *P* < 0.001), and learning atmosphere (β = 0.162, *P* < 0.05) had a significant positive impact on core competencies. The effects of course learning (*P* = 0.786), teacher-student relationships (*P* = 0.546), and institutional environment (*P* = 0.619) on core competencies were not statistically significant. See Table 4.

**Table 4:** Multiple linear regression analysis of core competencies.

Variable	Unstandardized coefficients		Standardized coefficients	*t*	Significance	VIF
	B	SE	β			
(Constant)	0.48	0.124		3.878	< 0.001	
Course learning	−0.014	0.053	−0.017	−0.272	0.786	4.4
Peer relationships	0.472	0.043	0.510	10.984	< 0.001	2.441
Learning atmosphere	0.138	0.067	0.162	2.045	0.042	7.134
Teacher-student relationships	0.037	0.062	0.044	0.604	0.546	5.96
Institutional environment	−0.03	0.06	−0.034	−0.498	0.619	5.403
Project practice	0.278	0.041	0.333	6.822	< 0.001	2.691

D-W = 2.085; R^2^ = 0.767; F = 144.708; *P* < 0.001.

## Discussion

In this study, public health students rated their overall core competencies relatively highly. The highest score in the *Responsibility and Accountability* category reflects their strong sense of social responsibility and cultural identity, this may be related to the professional mission emphasized in education (27). Relatively speaking, the *Humanistic Foundation* category scored the lowest (3.948 ± 0.589). This was particularly evident in items such as “reading humanistic books” and “understanding the humanistic spirit,” where performance was weaker. This finding suggests a deficiency in students' accumulation of humanistic knowledge and their theoretical understanding of the subject. This situation may be associated with the current state of medical education, which often features insufficient humanities coursework and an evaluation system that places greater emphasis on professional knowledge and technical skills (28). Public health education in China is still dominated by natural science courses, with humanities courses accounting for only 7.54% of the curriculum, which is far lower than the proportion in European and American countries (20%−26%) (29). Public health is a discipline that integrates both natural science and social-humanistic attributes (30). It requires not only solid professional skills but also profound humanistic care and ethical competence to face complex public health challenges. Therefore, strengthening the integration of humanities and social sciences content—such as literature, philosophy, and ethics—into the curriculum is essential. Promoting the deep integration of humanistic education with professional training is a key approach to enhancing students' comprehensive competencies (31).

This study found that students' awareness of core competencies had a significant impact on their competency scores. Specifically, students who reported understanding the content of core competencies scored significantly higher than those who had only heard of them or were unfamiliar with them. This finding suggests a link between students' level of awareness regarding core competencies and their self-assessed competency levels, as well as potential behavioral manifestations. Core competencies are not merely an accumulation of knowledge, they also integration of attitudes, values, and behavioral intentions (16). When students are familiar with this concept, it may enhance their capacity for self-reflection and goal orientation. As a result, they can more consciously practice these relevant competencies in their studies and daily life (32). Therefore, in public health education, greater emphasis should be placed on promoting and explaining the meaning of core competencies (33). These concepts should be integrated into orientation programs for new students, course introductions, or special topic lectures. This approach can help students establish a clear framework for competency development and guide them to actively enhance their overall competencies (34).

Multiple linear regression analysis revealed that peer relationships, project practice, and learning atmosphere—as dimensions of learning environment perception—had significant positive predictive effects on core competencies. Together, these factors explained 76.7% of the variance. Among them, peer relationships exerted the greatest influence, confirming the critical role of peer interaction in shaping values, fostering collaborative skills, and providing emotional support. This finding aligns with research by Wang (2024) conducted among midwifery students (35). Project practice highlights the importance of “learning by doing.” Public health is a highly practice-oriented discipline. Through hands-on activities such as internships, volunteer services, and research projects, students can translate theoretical knowledge into the ability to solve practical problems. This process further enhances their sense of innovation and social responsibility (36, 37). The learning atmosphere reflects the overall academic culture and collective motivation within the institution. A positive, open, and supportive atmosphere can help stimulate students' intrinsic motivation and spirit of inquiry.

It is worth noting that course learning, teacher-student relationships, and the institutional environment did not show significant effects in the regression analysis. This may suggest that, within the current educational framework, traditional curriculum design and patterns of teacher-student interaction have not yet been fully translated into effective enhancements of students' comprehensive competencies. Furthermore, if the institutional environment—such as teaching management and evaluation systems—is not deeply integrated with a competency-oriented approach, its educational effectiveness may also be limited (38). This calls for educators to further promote reforms in curriculum content and teaching methods. It is important to build a teaching environment that is more student-centered and places greater emphasis on process-oriented evaluation and feedback (39).

Based on the above findings, the following recommendations are proposed to enhance the core competencies of public health students. First, deepen the integration of humanistic education and professional education. This can be achieved by adding humanities and social sciences modules to the curriculum, encouraging interdisciplinary learning, and utilizing methods such as case-based teaching and narrative medicine to cultivate students' humanistic care and ethical judgment (40). Second, strengthen awareness-building and evaluative feedback regarding core competencies. Through workshops, competency development portfolios, and similar tools, help students understand the meaning of core competencies and their importance for professional growth. Incorporate competency indicators into the academic assessment system. Third, optimize the design of the learning environment by emphasizing practice and interaction. Establish cross-grade and cross-disciplinary collaborative learning platforms, expand practical training bases and project resources, and foster a supportive, inquiry-based learning atmosphere to stimulate students' initiative and innovation (30). Fourth, promote teacher development and teaching innovation. Enhance teachers' understanding of the core competency concept and their ability to translate it into teaching practices. Encourage the adoption of teaching models such as problem-based learning (PBL) and team-based learning to promote deeper interaction and common growth between teachers and students (34).

### Study limitations

This study has several limitations. First, it is a cross-sectional study with a sample drawn from a single institution, which may limit how well the findings apply to other settings. Second, data were collected using self-report scales, which may be influenced by social desirability bias. Furthermore, as a quantitative study, adding qualitative methods such as interviews would help provide deeper insight into the complex experiences of public health students. Future research could enlarge the sample by including students from multiple institutions and different regions. Combining qualitative interviews and behavioral observation to track how core competencies develop over time, as well as further examining how curriculum design, teaching strategies, and institutional arrangements specifically affect competency development, would be valuable next steps.

## Data Availability

The original contributions presented in the study are included in the article/supplementary material, further inquiries can be directed to the corresponding author.
